# Supplementary material for: The AFF-1 exoplasmic fusogen is required for endocytic scission and seamless tube elongation
Source: Nat Commun. 2018 May 1;9:1741. doi: 10.1038/s41467-018-04091-1 (PMC5931541; doi:10.1038/s41467-018-04091-1)
Supplement: Supplementary file 1 — Supplementary Information [file 41467_2018_4091_MOESM1_ESM.pdf]

Supplementary information for

The AFF-1 exoplasmic fusogen is required for endocytic scission and seamless tube elongation

<u>Authors</u>

Fabien Soulavie[1], David H. Hall[2] and Meera V. Sundaram[1]*

[1] Dept. of Genetics, University of Pennsylvania Perelman School of Medicine, Philadelphia, Pennsylvania, United States of America

[2]Department of Neuroscience, Albert Einstein College of Medicine, Bronx, New York, United States of America

***Author for correspondence**

446a Clinical Research Building

415 Curie Blvd.

Philadelphia PA 19104

sundaram@mail.med.upenn.edu

(215) 573-4527

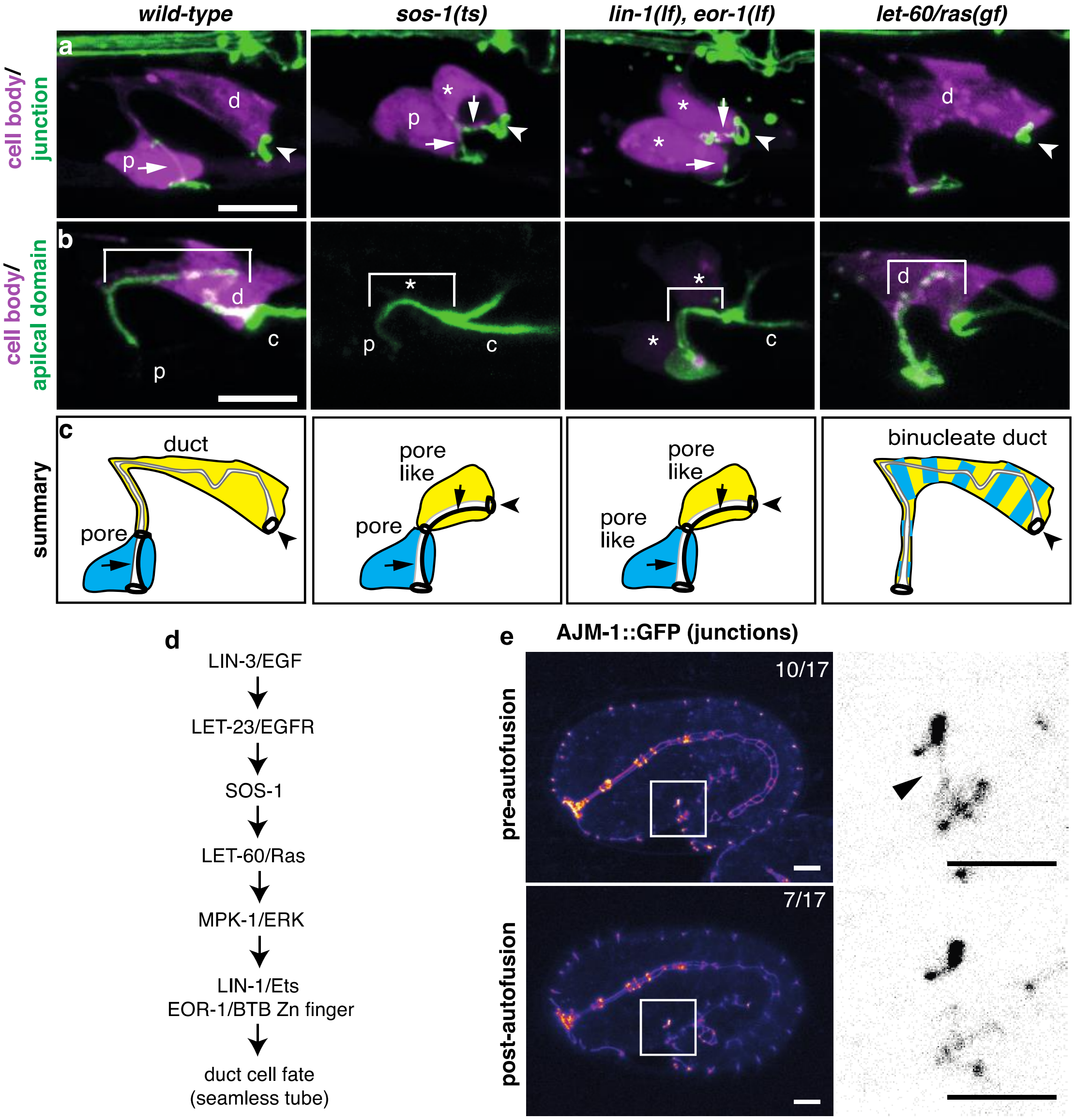

Supplementary Figure 1: EGF-Ras signaling promotes excretory duct fate, auto-fusion and shaping (**a and b**) Confocal Z-projections of duct and pore cells in early L1 larvae. **(a)** EGF-RAS-ERK signaling promotes auto-fusion. In magenta the duct (d) and pore (p) cell bodies with *dct-5pro::mCherry*, and in green the junction marker *ajm-1::GFP*. Arrows show cell auto-junctions, arrow-heads show duct-canal junctions. **(b)** EGF-RAS-ERK signaling promotes lumen elongation. In magenta the duct cell body with *lin-48pro::mRFP*, and in green the apical domain marker *rdy-2::GFP*. Brackets show duct lumen position. * = cell with altered fate. In Wild type, the duct lacks an auto-junction and has an elongated lumen. In the Ras pathway mutant *sos-1(cs41ts)* grown at restrictive temperature and in *lin-1(n304) eor-1(cs28)* double mutants, the two cells adopt a pore-like fate with auto-junctions and a short lumen. The duct cell body marker *lin-48pro::mCherry* is not expressed in *sos-1(cs41ts)* (**b**) at restrictive temperature and very weakly in *lin-1(n304) eor-1(cs28)* double mutant. In the constitutively active Ras mutant *let-60(n1046gf)*, the two cells fuse to make a binucleate duct-like cell without an auto-junction and with an elongated lumen. (**c**) schematics of phenotypes observed. **(d)** The canonical EGF-Ras-ERK signaling pathway leading to duct cell fate. (**e**) Confocal microscopy of two 1.5-fold stage embryos showing the cell junction marker *ajm-1*::GFP. Dorsal side up, anterior left in these and all subsequent images. At this stage, 10/17 embryos had a remaining duct auto-junction (upper panel) and 7/17 embryos showed no duct auto-junction (lower panel). Boxed regions are magnified in right panels. The arrow-head shows duct auto-junction. Scale bar = 5 μm.

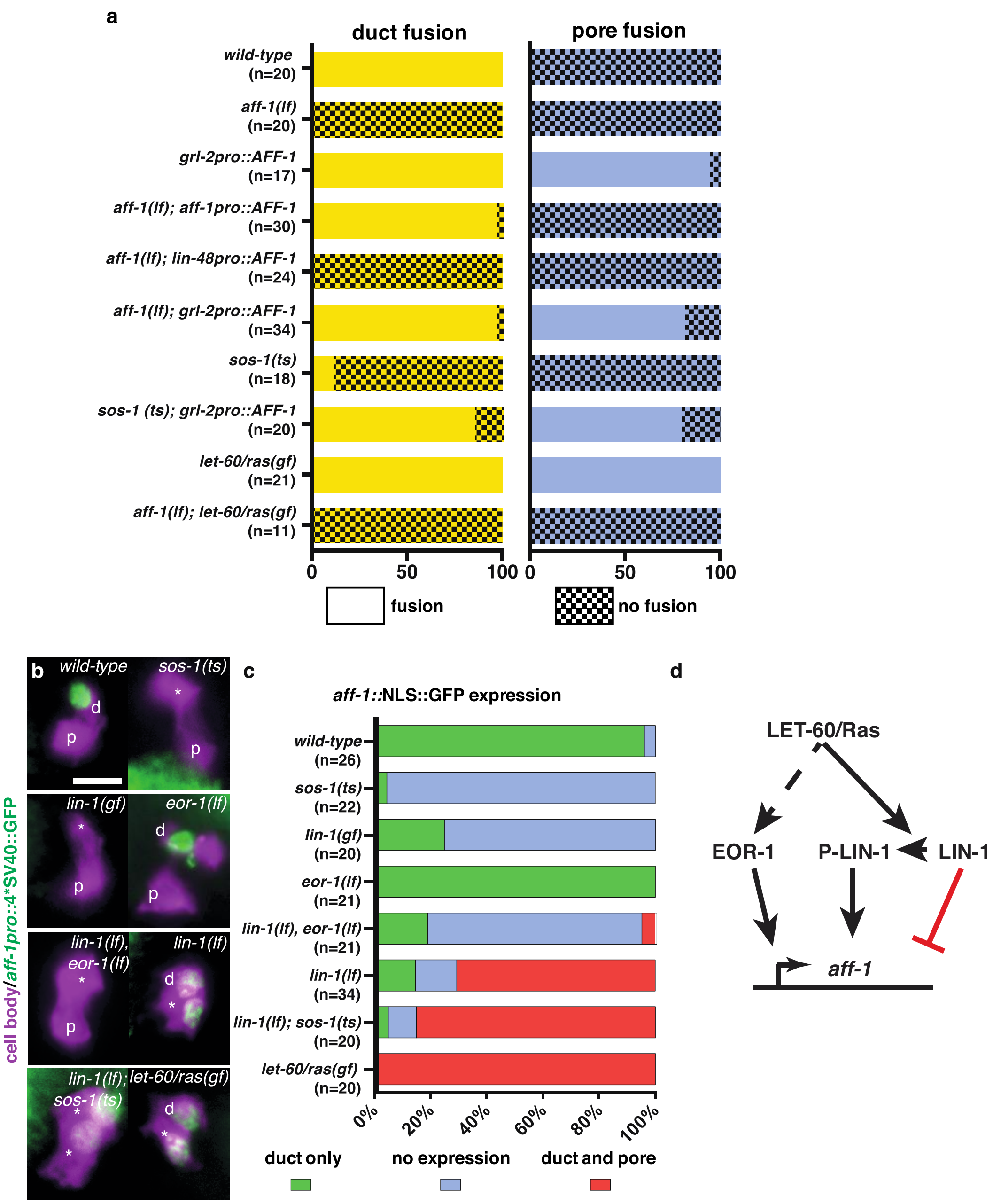

Supplementary Figure 2: Ras signaling promotes *aff-1* expression and auto-fusion through LIN-1 and EOR-1 Nuclear Factors. **(a)** Quantification of duct and pore auto-fusion in designated mutants. In WT, only the duct auto-fuses. In *aff-1(tm2214)* mutants, neither cell fuses. When AFF-1 is expressed in both cells with *grl-2pro*, both cells fuse, including in *aff-1(tm2214)* or the Ras signaling mutant *sos-1(cs41ts)*. The *aff-1(tm2214)* auto-fusion phenotype is rescued by expression of *aff-1* cDNA with a 5.4kb *aff-1* promoter. Later expression of *aff-1* with *lin-48pro* is not sufficient to rescue duct auto-fusion, suggesting there is a discrete window of time when auto-fusion can occur. Most Ras pathway mutants have cell fate transformations [1]; in these cases, duct and pore refer to the dorsal and ventral cells, respectively. Both cell fuse in the *let-60/Ras(n1046)* gain of function mutant, but the auto-fusions are abolished in the double mutant *aff-1(tm2214); let-60(n1046)*, showing that AFF-1 acts downstream of the Ras signaling pathway. For each cell, comparisons between genotypes were made by a one-tailed Fisher's Exact test and all relevant comparisons were p value < 0.0001. **(b)** 3-fold stage embryos of designated mutants expressing *aff-1pro::NLS-GFP*, showing the most common phenotype quantified in **(c).** d=duct cell; p=pore cell; * = cell with altered fate. **(d)** Model for *aff-1* transcriptional regulation by Ras signaling and the nuclear factors LIN-1 and EOR-1. P-LIN-1 is the ERK-phosphorylated form of LIN-1 [2]. Dashed arrow represents potential Ras-ERK-dependent regulation of EOR-1 [3,4]. Scale bar = 5 μm.

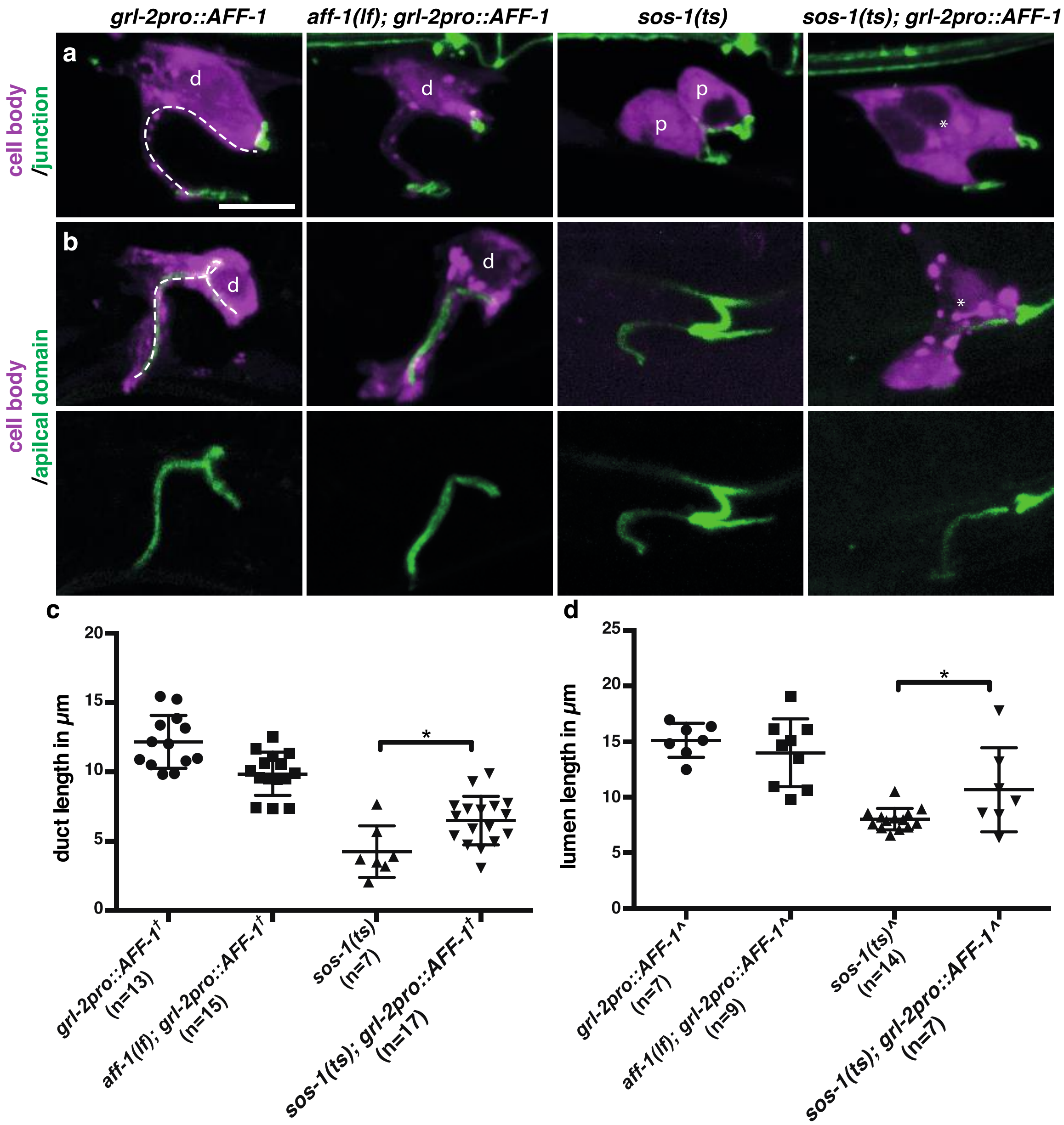

Supplementary Figure 3: AFF-1 is not sufficient to promote lumen elongation in Ras signaling mutants. (**a** and **b**) Confocal Z-projections of L1 stage larvae. Duct cell is labeled with *lin-48pro::mRFP* (*grl-2pro::AFF-1* and *aff-1(lf); grl-2pro::AFF-1*), *dct-5pro::mCherry* (*sos-1(ts)*) or *grl-2pro::mCherry* (*sos-1(ts); grl-2pro::AFF-1*) (magenta). In green, cell junctions are labeled with *ajm-1::GFP* in "**a**", or apical domains are labeled with *let-653::SfGFP* (*grl-2pro::AFF-1* and *aff-1(lf); grl-2pro::AFF-1*) or *rdy-2::GFP* (*sos-1(ts)* and *sos-1(ts); grl-2pro::AFF-1*) in "**b**". The excretory duct cell body and lumen have an elongated shape in *grl-2pro::AFF-1* or *aff-1(tm2214); grl-2pro::AFF-1*, but are shorter in *sos-1(cs41ts)* and *sos-1(cs41ts); grl-2pro::AFF-1*. (**c**) Measurements of duct cell length tracing from the pore-duct junction to the duct-canal junction, as indicated with the dotted line in panel "**a**" *grl-2::pro::AFF-1*. (**d**) Measurements of duct cell length, tracing as indicated with dotted line in panel "**b**" *grl-2::pro::AFF-1*. †=measure on a binucleate cell. ^=duct and pore lumen. Error bars = ± standard deviation (SD). Scale bar = 5 μm. *= *p-value* < 0.05, Mann-Whitney test.

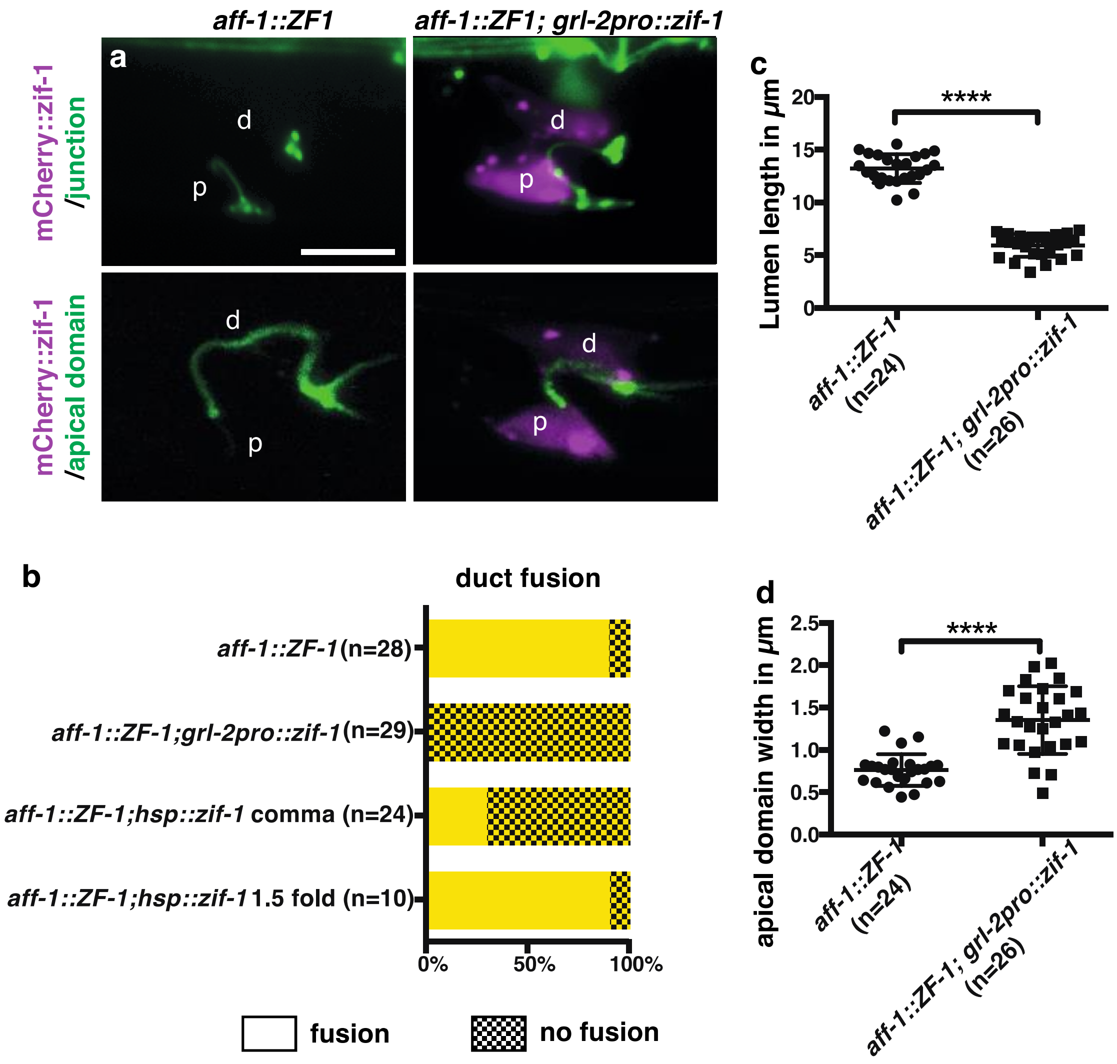

Supplementary Figure 4: Early conditional knock-down of *aff-1* in the duct and pore prevents excretory duct auto-fusion and tube elongation. (**a**) Confocal Z-projections of L1 stage larvae, d, duct; p, pore. The cell junctions are labeled in green with *ajm-1::GFP* (upper panels) or apical domains are labeled in green with *rdy-2::GFP* (lower panels) and the ZIF-1::mCherry expression pattern is labeled in magenta. The fusion protein AFF-1::ZF1 expressed by the CRISPR-Cas9 gene edited allele *aff-1(cs232, [aff-1::zf1])* is able to drive excretory duct auto-fusion and lumen elongation (left panels**)** and can be targeted for early degradation by the protease ZIF-1 expressed with *grl-2pro::zif-1::mCherry* (right panels**)** to prevent auto-fusion and lumen elongation. **b** shows quantification of the duct fusion phenotype in *aff-1 (cs232, [aff-1::zf1])* animals with various ZIF-1-expressing transgenes, including heat-shock promoter (hsp)-driven expression before (comma stage) and after (1.5-fold stage) the normal time of duct auto-fusion. Comparisons were made by one-tailed Fisher's Exact test, and all the relevant comparisons were p value < 0.0001. **c** and **d** show quantification of duct lumen length and apical domain width respectively. ****= *p-value* < 0.0001. Mann-Whitney test. Error bars = ± SD. Scale bar = 5 μm.

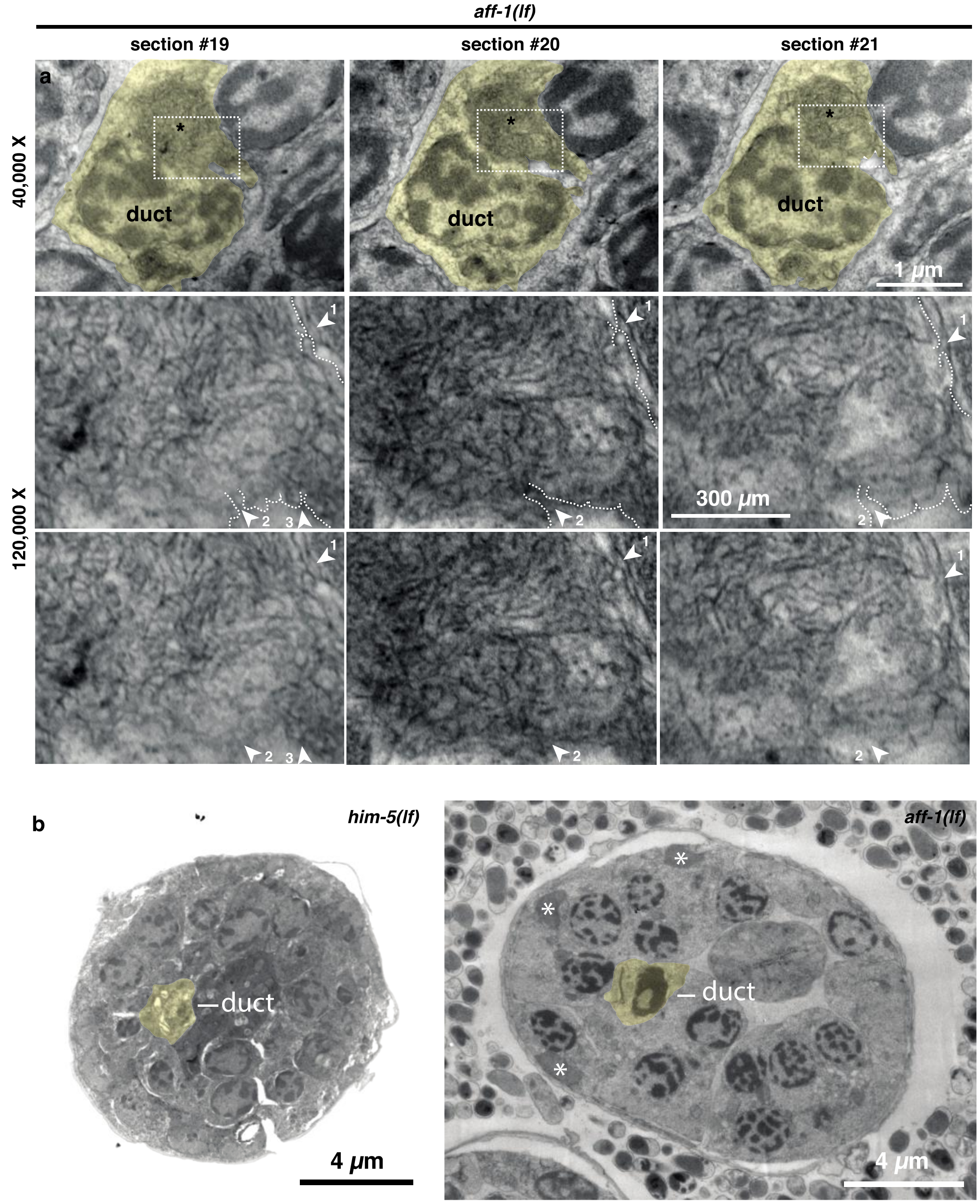

Supplementary Figure 5: *aff-1* mutants accumulate membrane inclusions connected to the basal plasma membrane. (**a**) Serial TEM sections of the *aff-1(tm2214)* L1 larva shown in Figure 5a. The upper panels show a 40,000X magnification (with duct cell post-colored in yellow), and the middle and lower panels show the boxed region at 120,000X magnification. A large membrane inclusion (*) can be observed adjacent to the cell edge. In the middle panels, the dotted lines show the position of the basal plasma membrane, and the arrowheads show three positions where the basal plasma membrane invaginates into the large membrane inclusion. The lower panels are identical to the middle panels without membrane indication. (**b)** TEM transverse slices of normal (*him-5(e1490)*) or *aff-1(tm2214)* L1 duct (post-colored in yellow). Asterisks indicate membrane inclusions in hypodermal cells.

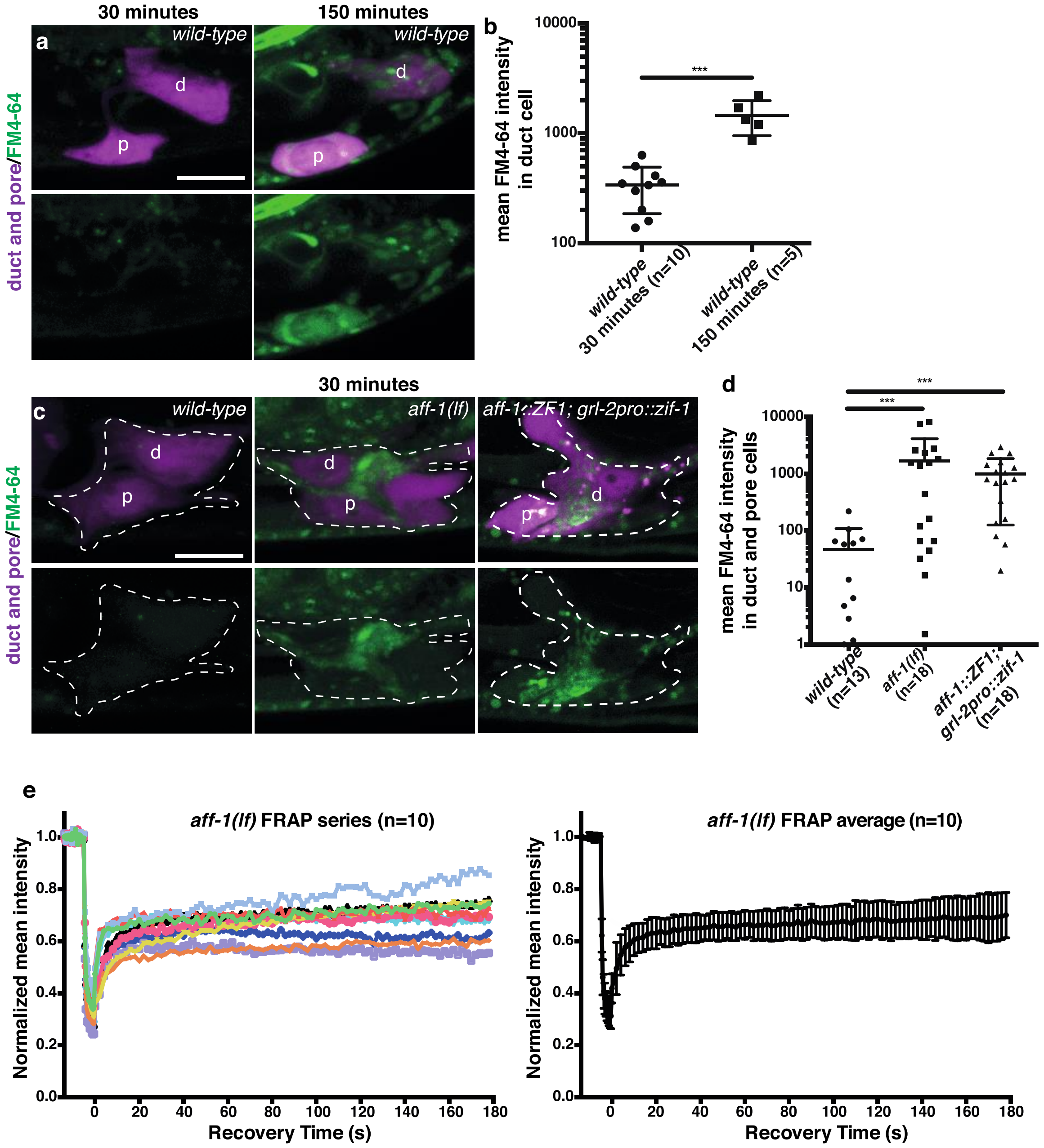

Supplementary Figure 6: The duct cell accumulate lipid binding dyes. a. L1 larvae. **c**. L4 larvae. **a** and **c**. Confocal Z-projections. In magenta the duct (d) and pore (p) cell bodies with *grl-2pro::YFP*, and in green FM4-64 dye. (**a** and **b**) *Wild-type* L1s have little or no dye penetration after a 30 minute exposure + 30 minute recovery and a moderate penetration after a 150 minute exposure + 30 minute recovery, demonstrating endocytic activity in these cells. Scale bar = 5 μm. (**c** and **d**) *Wild-Type* L4s have little or no dye penetration after a 30 minute exposure + 30 minute recovery, but significant penetration is observed in *aff-1(tm2214)* mutants or in the duct and pore-specific *aff-1* knock down [*aff-1(cs232, [aff-1::zf1]); grl-2pro::zif-1*. Scale bar = 10 μm. **b** and **d** show quantification of dye fluorescence intensity in the duct, or in both the duct and pore, respectively. Note logarithmic scale. Some data points were "0" could not be shown on this scale. ***= *p-value* < 0.001, Mann and Whitney test. (**e**) Time lapse of Fluorescence Recovery After Photo-bleaching as shown in Figure 5d, showing the fluorescence recovery of FM4-64 in *aff-1(tm2214)* mutants. The left panel shows the complete set of data on 10 L1 larvae. The right panel shows the average and the standard deviation of the same set of data. Error bars = ± SD.

Supplementary **Table 1.**

C. elegans strains used in this work

strain name	full genotype
AD281	*him-5(e1490) V*
BP601	*aff-1(tm2214)/mIn1 II*
SU93	*jcIs1(ajm-1::gfp,rol-6) IV*
UP2030	*let-60, jcIs1(ajm-1::gfp,rol-6) IV*
UP2042	*jcIs1(ajm-1::gfp,rol-6) IV;csEx256(lin-48::mRFP,unc-119)*
UP2118	*unc-119 III;jcis-1(ajm-1::gfp, rol-6) IV;qnEx-59(dct-5p::mRFP), unc-119+*
UP2314	*mcEx337(VHA-5::GFP, rol-6d)*
UP2369	*jcIs1(ajm-1::gfp,rol-6) IV;sos-1(cs41) V;qnEx59(dct-5p::mRFP)*
UP2369	*jcIs1(ajm-1::gfp,rol-6) IV;sos-1(cs41) V;qnEx59(dct-5p::mRFP)*
UP2594	*aff-1(tm2214)/mIn1 II;jcIs1(ajm-1::gfp,rol-6) IV;CsEx256(lin-48::mRFP)*
UP2595	*aff-1(tm2214)/mIn1 II;jcIs1(ajm-1::gfp,rol-6)IV*
UP2659	*aff-1(tm2214)/mIn1 II;jcIs1(ajm-1::gfp,rol-6)IV;CsEx419(lin-48::aff-1)*
UP2684	*csEx444 (let-653p::LET-653-sfGFP, lin-48p::mRFP)*
UP2726	*aff-1(tm2214)/mIn1 II; jcIs1(ajm-1::gfp,rol-6)IV; csEx451 (grl-2p::aff-1, myo-2::mRFP)*
UP2834	*aff-1(tm2214)/mIn1 II; csEx444 (let-653p::LET-653-SF-GFP, lin-48p::mRFP)*
UP2874	*aff-1(tm2214)/mInI II; jcIsI(ajm-1::gfp,rol-6) IV; csEx539 (aff-1p::AFF-1, myo-2p::mRFP)*
UP2883	*csIs62 (aff-1p::4*SV40::GFP, grl-2p::mRFP)*
UP2907	*csIs62 (aff-1p::4*SV40::GFP, grl-2p::mRFP); lin-1 (n304)/nT1g[qIs51] IV*
UP2908	*csIs62 (aff-1p::4*SV40::GFP, grl-2p::mRFP)/+; lin-1 (n304), eor-1(cs28)/nT1g[qIs51] IV*
UP2909	*csIs62 (aff-1p::4*SV40::GFP, grl-2p::mRFP); lin-1 (n1761)/nT1g[qIs51] IV*
UP2925	*csIs61(RDY-2::GFP, lin-48pro::mRFP)*
UP2931	*jcIs1(ajm-1::gfp,rol-6) IV; sos-1(cs41,ts) V; csEx453 (grl-2p::aff-1, myo-2p::mRFP)*
UP2963	*csIs62 (aff-1p::4*SV40::GFP, grl-2p::mRFP); jcis1, eor-1(cs28) IV*
UP2964	*sos-1(cs41) V; csIs62 (aff-1p::4*SV40::GFP, grl-2p::mRFP)*
UP3008	*aff-1(tm2214) II;csEx585(aff-1p::AFF-1, lin-48p::mRFP, lin-48p::LET-653::sfGFP)*
UP3024	*csEx587(grl-2p::AFF-1, lin-48p::mRFP, lin-48p::LET-653::sfGFP)*
UP3053	*aff-1(tm2214)/mIn1 II; jcIs1(ajm-1::gfp,rol-6) IV; csEx585(aff-1p::AFF-1, lin-48p::mRFP, lin-48p::LET-653::sfGFP)*
UP3078	*aff-1(tm2214)/mIn1 II; let-60(n1046), jcIs1(ajm-1::gfp,rol-6) IV*
UP3120	*lin-1(n304) IV; sos-1(cs41) V; csIs62(aff-1p::4*SV40::GFP, grl-2p::mRFP)*
UP3161	*lin-1(n304) eor-1/nT1[qIs51] IV; csIs61 (RDY-2::GFP, lin-48pro::mCherry)*
UP3176	*jcIs1(ajm-1::gfp,rol-6) IV;sos-1(cs41) V;CsEx677(grl-2pro::mRFP, grl-2pro::AFF-1))*
UP3177	*lin-1(n304), eor-1(cs28)/nT1[qIs51] IV;wIs78 X; qnEx59(dct-5pro::mRFP)*

Supplementary **Table 1.** (Continued)

strain name	full genotype
UP3183	*aff-1(tm2214)/mIn1 II; jcIs1(ajm-1::gfp,rol-6), let-60(n1046) IV; csEx444 (let-653p::LET-653-SF-GFP, lin-48p::mRFP)*
UP3186	*jcIs1(ajm-1::gfp,rol-6) IV; csEx672(grl-2pro::AFF-1, lin-48p::mRFP)*
UP3187	*let-60(n1046) IV; csEx444(let-653p::LET-653-SF-GFP, lin-48p::mRFP)*
UP3188	*let-60(n1046) IV; csIs62 (aff-1p::4*SV40::GFP, grl-2p::mRFP)*
UP3196	*let-60(n1046), jcIs1(ajm-1::gfp,rol-6) IV; csEx256(lin-48pro::mCherry)*
UP3199	*sos-1(cs41) V; csEx680(grl-2pro::AFF-1, grl-2pro::mRFP; RDY-2::GFP)*
UP3206	*aff-1(tm2214)/mIn1 II; mcEx337(vha-5::GFP, rol-6)*
UP3207	*aff-1(tm2214)/mIn1 II; jcIs1(ajm-1::gfp,rol-6), let-60(n1046) IV; csEx256 (lin-48p::mRFP)*
UP3430	*csEx813(grl-2pro::YFP)*
UP3431	*csEx814 (aff-1pro::aff-1::mCherry, grl-2pro::YFP)*
UP3476	*aff-1(cs232, [aff-1::ZF1, sqt-1(d), hs::cre, hygroR]) II; jcIs1(ajm-1::gfp,rol-6) IV; csEx749 (grl-2pro ::zif-1, CC ::GFP)*
UP3486	*aff-1(cs232, [aff-1::ZF1, sqt-1(d), hs::cre, hygroR]) II; rdy-2 (cs233,[rdy-2::GFP]); csEx749 (grl-2pro ::zif-1, CC ::GFP) V*
UP3488	*aff-1(cs232, [aff-1::ZF1, sqt-1(d), hs::cre, hygroR]) II; rdy-2 (cs233,[rdy-2::GFP]) V*
UP3521	*aff-1(cs232, [aff-1::ZF1, sqt-1(d), hs::cre, hygroR]) II; rdy-2 (cs233,[rdy-2::GFP]) V; csEx847(hsp::zif-1::mCherry, ajm-1::mCherry)*
UP3532	*let-60(n1046) IV; csIs61(RDY-2::GFP, lin-48pro::mRFP)*
UP3538	*rab-11(tm2063) I/hT2[bli-4(e937) let-?(q782) qIs48]; rdy-2 (cs233,[rdy-2::GFP]) V*
UP3558	*dyn-1(ky51ts) X; rdy-2 (cs233,[rdy-2::GFP]) V*
UP3564	chc-1(b1025ts) III/hT2[bli-4(e937) let-?(q782) qIs48]; rdy-2 (cs233,[rdy-2::GFP]) V
UP3563	rab-5 (ok2605) I/*hT2[bli-4(e937) let-?(q782) qIs48]*; rdy-2 (cs233,[rdy-2::GFP]) V

Supplementary **Table 2.**

Transgenes used in this work

Name	Description	Type	Construction
csEx527	*aff-1p::4*SV40::GFP, grl-2pro::mCherry*	multi-copy external chromosomal array	Injection of pFS115 (30 ng μL^{-1}) and pFS107 (30 ng μL-1)
csIs62	*aff-1p::4*SV40::GFP, grl-2pro::mCherry*	multi-copy Inserted	Insertion of *csEx527*
csEx680	*grl-2pro::AFF-1, grl-2pro::mRFP; RDY-2::GFP*	multi-copy external chromosomal array	Injection of pFS108 (30 ng μL-1) and pCFJ90 (2 ng μL-1)
csEx539	*aff-1pro::AFF-1, myo-2p::RFP*	multi-copy external chromosomal array	Injection of pFS122 (30 ng μL-1) and pCFJ90 (2 ng μL-1)
csEx419	*lin-48pro::AFF-1, myo-2p::RFP*	multi-copy external chromosomal array	Injection of pFS102 (30 ng μL-1) and pCFJ90 (2 ng μL-1)
csEx451	*grl-2pro::aff-1, myo-2p::RFP*	multi-copy external chromosomal array	Injection of pFS108 (30 ng μL-1) and pCFJ90 (2 ng μL-1)
csEx585	*aff-1pro::AFF-1, lin-48p::mCherry, lin-48p::LET-653::sfGFP*	multi-copy external chromosomal array	Injection of pFS122 (30 ng μL-1), pJAF2 (5 ng/μl) and pHS4 (50 ng μL-1)
csEx587	*grl-2p::AFF-1, lin-48p::mCherry, lin-48p::LET-653::sfGFP*	multi-copy external chromosomal array	Injection of pFS108 (30 ng μL-1) and pJAF2 (5 ng μL-1) and pHS4 (50 ng μL-1)
csEx742	*grl-2pro::mCherry*	multi-copy external chromosomal array	Injection of pFS107 (30 ng μL-1)
csEx749	*grl-2pro ::zif-1, CC ::GFP*	multi-copy external chromosomal array	Injection of pFS133 (30 ng μL-1) and CC::GFP ? (30 ng μL-1)
csEx740	*hsp-16-41pro::zif-1::mCherry, myo-2::GFP*	multi-copy external chromosomal array	Injection of pSA120 (30ng μL-1) and *myo-2::GFP* (4 ng μL-1)
csEx847	*hsp-16-41pro::zif-1::mCherry, ajm-1::mCherry*	multi-copy external chromosomal array	Injection of pSA120 (30 ng μL-1) and pKM15 (30 ng μL-1)
csEx814	*aff-1pro::aff-1::mCherry, grl-2pro::YFP*	multi-copy external chromosomal array	Injection of pFS145 (5 ng μL-1) and pFSKM15 (20 ng μL-1)
cs232	*aff-1(cs232, [aff-1::ZF1, sqt-1(d), hs::cre, hygroR])*	CRISPR-Cas9 generated allele	See materials and methods
cs233	*rdy-2(cs233, [rdy-2::gfp])*	CRISPR-Cas9 generated allele	See materials and methods

Supplementary **Table 3.**

PCR primers used in this work

Name	Sequence
oFS-5	tgtaccGGTACCaaaatgcgactgtggcaatggt
oFS-6	tgtaccGCTAGCttagtaatcagatgaattcttc
oFS-7	tgtaccGCTAGCaaaatgcgactgtggcaatggt
oFS-8	tgtaccGGTACCttagtaatcagatgaattcttc
oFS-23	gggaaaGGTACCATCGAGTGCGTCAGATTAGC
oFS-24	gggaaaGATATCGCTCAGTTGAGGCCTATCG
oFS-45	tgtaccgtcgacGGATGAGACAAAAGAAGATTG
oFS-46	tgtaccCCCGGGCTGAAATTAAATAATTATAGGC
oFS-64	cgctACCGGTagctCCGCGGgtaatcagatgaattcttcttt
oFS-69	tgtaccGCTAGCaaaATGAGTGAGTGTTCCGCGAGTACC
oFS-70	ggacCTACTTATACAATTCATCCATGCC
oFS-130	GttactaaaagctcattcacaGTTTTAGAGCTAGAAATAGCAAGT
oFS-131	GAAAAATGCAGGAAAGTAAGTGTTTTAGAGCTAGAAATAGCAAGT
oFS-140	TGCGCCGCGGATGGTCTCAAAGGGTGAAGAAG
oFS-141	CGACACCGGTCTACTTATACAATTCATCCATGCC
oFS-142	cggccagtcgccggcagctcaatgggtgtgtattcttccc
oFS-143	TCGCGTTTTGTATTCTGTgtaatcagatgaattcttcttttc
oFS-144	tgattacACAGAATACAAAACGCGACTTTG
oFS-145	GTACAGATTCTCttaCCTCGGAACTCTCAGCTCATC
oFS-146	GTTCCGAGGtaaGAGAATCTGTACTTTCAATCCGG
oFS-147	gtgtgaatgagcttATAACTTCGTATAATGTATGCTATACG
oFS-148	TACGAAGTTATaagctcattcacacggtgaatcc
oFS-149	cagctatgaccatgttataaaatcctttaaggcacgcc
oFS-150	ggattttataacatggtcatagctgtttcctg
oFS-151	tgagctgccggcgactggccgtcg
oFS-167	acgttgtaaaacgacggccagtcgccggcaGATGGGCAGTTGCGGCGg
oFS-168	CATCGATGCTCCTGAGGCTCCCGATGCTCCAAATGAAGATCGGATGGTACG
oFS-169	CGTGATTACAAGGATGACGATGACAAGAGATGAatgaattccaaattcccaagaaatcga
oFS-170	ggaaacagctatgaccatgttatcgatttcTCGTCGCTACACATCATGCC
oFS-171	ggtgagtgcacgttgtttcg
oFS-172	gtttgatctacaggtattgcgg
oFS-173	gatcaaacggtgagtgcacgGTTTTAGAGCTAGAAATAGCAAGT

Supplementary **Table 4.**

DNA plasmids used in this work

Name	description	backbone	method
pFS127	*aff-1pro* empty	pPD49.26	*aff-1* promoter PCR amplified from WRM0615dE03 with primers oFS-45 and oFS-46 and cloned into pPD49.26 (Addgene) as a SalI/XmaI digestion product
pFS115	*aff-1pro*::4*NLS ::GFP	pPD49.26	4*SV40::GFP was PCR amplified from pPD121.83 (Addgene) with primers oFS-130 and oFS-131, and inserted as a Blunt Ended/NheI fragment in pFS127
pFS102	*lin-48pro::aff-1*	pPD49.26	*aff-1* cDNA PCR amplified from pIZT::aff-1 with primers oFS-7 and oFS-8 and cloned into pJP49 as a NheI/KpnI digestion product
pFS108	*grl-2pro::aff-1*	pPD49.26	*aff-1* cDNA PCR amplified from pIZT::aff-1 with primers oFS-7 and oFS-8 and cloned into pJC2 as a NheI/KpnI digestion product
pFS122	*aff-1pro::aff-1*	pPD49.26	*aff-1* cDNA PCR amplified from pIZT::aff-1 with primers oFS-7 and oFS-8 and cloned into pFS127 as a NheI/KpnI digestion product
pFS145	*aff-1pro::AFF-1::mCherry*	pPD49.26	PCR amplification of *aff-1* cDNA without stop codon by oFS-7 and oFS-64 and in frame addition of *mCherry* coding sequence, PCR amplified from pSA120 (Addgene) with oFS-140 and oFS-141
pFS107	*grl-2pro::mCherry*	pPD49.26	PCR amplification of *grl-2pro* from pKM15 with oMS-201 and oMS-203, and clone as Sph1-BamH1 fragment into pPD49.26 to obtain pJC2, and *mCherry* coding sequence was PCR amplified from pMH4 (Max Heiman) with oFS-23 and oFS-24 and was inserted as KpnI-EcoRV
pFS133	*grl-2pro::zif-1::mCherry*	pPD49.26	PCR amplification of *zif-1::mCherry* from pSA120 with oFS-69 and oFS-70 and inserted into the pPD49.26 derivatives, pJC2 (*grl-2pro*)
pFS135	*lin-48pro::zif-1::mCherry*	pPD49.26	PCR amplification of *zif-1::mCherry* from pSA120 with oFS-69 and oFS-70 and inserted into the pPD49.26 derivatives, pJP49 (*lin-48pro*)
pFS144	Peft-3::Cas9 + aff-1 sgRNA	pDD162	See material and methods for full description
PRFR55	Peft-3::Cas9 + rdy-2 sgRNA	pDD162	See material and methods for full description
pFS146	AFF-1 repair templates + ZF1^SEC^STOP	pDD282	See material and methods for full description
pRFR56	RDY-2 repair templates + GFP^SEC^3xFlag	pDD282	See material and methods for full description

Supplementary references:

1. Abdus-Saboor I*, et al.* Notch and Ras promote sequential steps of excretory tube development in C. elegans. *Development* **138**, 3545-3555 (2011).

2. Jacobs D, Beitel GJ, Clark SG, Horvitz HR, Kornfeld K. Gain-of-function mutations in the Caenorhabditis elegans lin-1 ETS gene identify a C-terminal regulatory domain phosphorylated by ERK MAP kinase. *Genetics* **149**, 1809-1822 (1998).

3. Howard RM, Sundaram MV. C. elegans EOR-1/PLZF and EOR-2 positively regulate Ras and Wnt signaling and function redundantly with LIN-25 and the SUR-2 Mediator component. *Genes Dev* **16**, 1815-1827 (2002).

4. Howell K, Arur S, Schedl T, Sundaram MV. EOR-2 is an obligate binding partner of the BTB-zinc finger protein EOR-1 in Caenorhabditis elegans. *Genetics* **184**, 899-913 (2010).
